# Paramylon extracted from *Euglena gracilis* EOD-1 augmented the expression of SIRT1

**DOI:** 10.1007/s10616-021-00494-z

**Published:** 2021-09-15

**Authors:** Hiromi Ieiri, Natsuki Kameda, Junko Naito, Takanori Kawano, Norihisa Nishida, Madoka Takahashi, Yoshinori Katakura

**Affiliations:** 1 Graduate School of Bioresources and Bioenvironmental Sciences, Kyushu University, 744 Motooka, Nishi-ku, Fukuoka, 819-0395 Japan; 2 Graduate School of Systems Life Science, Kyushu University, 744 Motooka, Nishi-ku, 819-0395 Fukuoka, Japan; 3 Kobelco Eco-Solutions Co. Ltd., Kobe, Hyogo 651-2241 Japan; 4 Faculty of Agriculture, Kyushu University, 744 Motooka, Nishi-ku, Fukuoka, 819-0395 Japan

**Keywords:** SIRT1, HaCaT, Caco-2, Gut–skin interaction, Microarray

## Abstract

*Euglena gracilis*, a type of microalgae, contains several nutrients and accumulates paramylon, a β-1,3-glucan. In recent studies, paramylon has shown to exhibit various activities including immunomoduratory and hepatoprotective effects. In the present study, using an in vitro cell culture system, we aimed to determine whether paramylon derived from the *E. gracilis* EOD-1 strain, which produces large amounts of paramylon, can augment SIRT1 expression in epidermal cells via activating gut–skin interactions. Results showed that paramylon augmented the expression of SIRT1 in Caco-2 cells, a human intestinal cell line. Furthermore, microarray analysis of Caco-2 cells treated with paramylon showed that paramylon activates epidermal cells through inducing the secretion of factors from intestinal cells. Then, we focused on skin cells as target cells of paramylon-activated intestinal cells. Results showed that secretory factors from Caco-2 cells treated with paramylon augmented the expression of SIRT1 in HaCaT cells, a human keratinocyte cell line, and that expression level of genes related to the growth and maintenance of epidermal cells were significantly changed in Caco-2 cells treated with paramylon as evidenced by microarray analysis. All these results suggest that paramylon can activate epidermal cells by inducing the production of secretory factors from intestinal cells.

## Introduction

The unicellular phototrophic protist *Euglena gracilis* is ubiquitously present in most freshwater biotopes. *E. gracilis* accumulates of β-1,3-glucan, paramylon (Malkoff and Buetow 1964; Gissibl et al. 2019). paramylon is of special interest because of its immunomodulatory effects on human lymphocytes (Russo et al. 2017; Barsanti and Gualtieri 2019), inhibitory effects on fibrosis and non-alcoholic steatohepatitis (Nakashima et al. 2019), and hepatoprotective effects (Sugiyama et al. 2009). In particular, the *E. gracilis* EOD-1 strain produces high paramylon yields (70–80%) (Ishibashi et al. 2019). Oral administration of *E. gracilis* EOD-1 strain has been reported to increase sIgA concentration in saliva (Ishibashi et al. 2019). Oral administration of paramylon purified from the EOD-1 strain ameliorate impaired glucose tolerance and lower serum lipid levels in diet-induced obesity mouse models (Aoe et al. 2019). In the present study, we aimed to clarify the systemic anti-aging effects of paramylon via activating intestinal cells, and possible improvement of skin through the activation of gut–skin interaction. The interaction between the gut and the skin and its molecular basis has been studied in relation to the intestinal tract and skin wounds of fish, but very few studies have actually been done (Mateus et al. 2021). In this study, we also aim to analyze the interaction between the intestinal tract and the skin, and the possibility of its regulation by food.

## Materials and methods

### Paramylon extraction

Paramylon derived from *E. gracilis* EOD-1 was prepared as described previously (Aoe et al. 2019).

### Cell lines

A human colon cancer cell line, Caco-2, and a human keratinocyte cell line, HaCaT were cultured in Dulbecco’s modified Eagle’s medium (DMEM; Nissui, Tokyo, Japan) supplemented with 10% fetal bovine serum (FBS; Capricorn Scientific, Germany) at 37 °C in a 5% CO_2_ atmosphere.

### Evaluation of the effect of paramylon on SIRT1 promoter

HaCaT cells transduced with the EGFP expression vector under the control of human SIRT1 promoter (HaCaT (SIRT1p-EGFP)) were treated with conditioned media from Caco-2 cells treated with paramylon. Changes in EGFP fluorescence derived from pSIRT1p-EGFP were monitored with an IN Cell Analyzer 2200 (GE Healthcare, Little Chalfont, UK) (Chong et al. 2019).

### Co-culture system

Caco-2 cells were seeded onto permeable supports in 24-well plates with 0.4 μm transparent PET membranes (Falcon, NY, USA), and cultured for 15 days. The transepithelial electrical resistance value was measured using a Millicell ERS-2 (Merck Millipore), and if the value was > 1000 Ω cm^2^, 100 µg/mL paramylon was added to the Caco-2 cells (apical side). HaCaT cells (basolateral side) and Caco-2 cells (apical side) were co-cultured for 48 h.

### Quantitative reverse transcription PCR (qRT-PCT)

Total RNA was extracted using the High Pure RNA Isolation kit (Roche Diagnostics, GmbH, Manheim, Germany), and reverse transcribed into cDNA using RevrerTra Ace (Toyobo, Osaka, Japan). qPCR was performed using the Thunderbird SYBR qPCR mix (Toyobo) in a Thermal Cycler Dice Real Time System TP-800 (Takara, Shiga, Japan). The following qPCR primer sequences were used: β-actin forward, 5′-TGGCACCCAGCACAATGAA-3′ and reverse, 5′-CTAAGTCATAGTCCGCCTAGAAGC-3′; SIRT1 forward, 5′-GCCTCACATGCAAGCTCTAGTGAC-3′ and reverse, 5′-TTCGAGGATCTGTGCCAATCATAA-3′ (Harada et al. 2016).

### Microarray

Total RNA was isolated from Caco-2 cells treated with 100 µg/mL paramylon or PBS, using Isogen II (Nippon Gene, Tokyo, Japan). cRNA was labeled with Low Input Quick Amp Labeling (Agilent Technologies, Santa Clara, CA, USA), and hybridized to DNA microarray (SurePrint G3 Human Gene Expression 8 × 60 K v3, Agilent). Relative hybridization intensities and background hybridization values were calculated using Feature Extraction Software (Agilent) (Sugihara et al. 2019).

We identified genes with altered expression, as described previously (Bolstad et al. 2003). We then established criteria for significantly up- or down-regulated genes: up-regulated genes, Z-score ≥ 2.0 and ratio ≥ 1.5-fold; down-regulated genes: Z-score ≤ − 2.0 and ratio ≤ 0.66-fold. Significantly over-represented GO categories were determined as described above.

### Statistical analysis

All experiments were performed at least 3 times, and the corresponding data are shown. The results are presented as man ± standard deviation. Multiple comparisons between groups were made by one-way ANOVA with Turkey’s post hoc test. Statistical significance was defined as P < 0.05 (*P < 0.05; **P < 0.01; ***P < 0.001).

## Results and Discussion

**Fig. 1:**
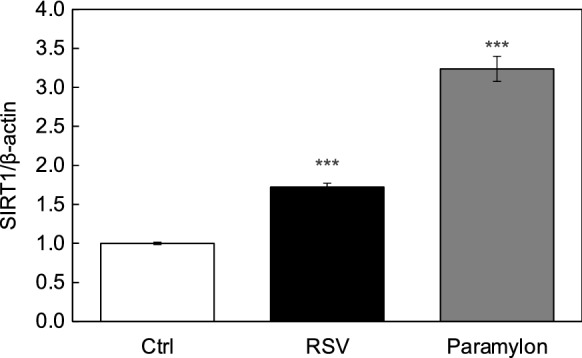
paramylon augmented SIRT1 expression in Caco-2 cells. Caco-2 cells were treated with 100 µg/mL paramylon and cultured for 2 days. The effect of paramylon on the expression of SIRT1 in Caco-2 cells were evaluated by qRT-PCR. Statistical significance was determined using a two-sided Student’s *t *test. Statistical significance was defined at P < 0.05 (***P < 0.001)

To clarify whether orally ingested paramylon induces systemic ant-aging, we firstly investigated whether paramylon affects gene expression of anti-aging gene, SIRT1, in human intestinal cell line, Caco-2 cells. As shown in Fig. 1, 100 µg/mL paramylon greatly augmented the expression of SIRT1 as comparable to resveratrol (10 µM), where resveratrol was used as a positive control for the augmented expression of SIRT1 (Chong et al. 2019). This result suggest that paramylon activate intestinal cells. To elucidate the molecular mechanisms underlying the systemic effects of paramylon through the activation of intestinal cells, we performed a microarray assay to detect significantly up- and down-regulated genes in Caco-2 cells following the treatment with paramylon. Genes relating to epidermal (4 genes) or dermal (6 genes) function, calcium regulation (45 genes), collagen synthesis (9 genes), the β-catenin pathway (15 genes) and secretory factors (11 genes) were significantly up- or down-regulated genes in Caco-2 cells treated with paramylon (Table 1). These results suggest that paramylon activate systemic cells through inducing the secretion of factors.

**Fig. 2:**
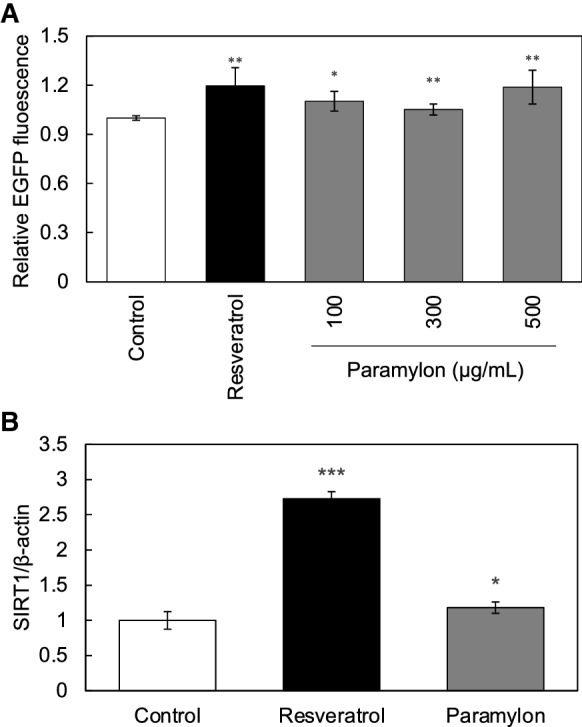
paramylon activated skin SIRT1 production via gut–skin interaction. **A** Effect of conditioned media from Caco-2 cells treated with paramylon on SIRT1 expression in HaCaT cells was evaluated by using IN Cell Analyzer 2200. **B** Effect of secreted factors from Caco-2 cells treated with paramylon on the expression of endogenous SIRT1 in HaCaT cells, assessed by using an in vitro co-culture system, was evaluated by qRT-PCR. In both experiments, resveratrol (10 µM) was added directly to HaCaT cells and effects on the expression of SIRT1 were evaluated by using IN Cell Analyzer 2200 and qRT-PCR

We then focused on skin cells as target cells of paramylon-activated intestinal cells. Firstly, we investigated whether conditioned media from cultured Caco-2 cells treated with paramylon could activate skin cells. To this end, conditioned media from Caco-2 cells treated with 100 to 500 µg/mL paramylon was collected, and HaCaT (SIRT1p-EGFP) cells were cultured in this medium. As shown in Fig. 3 A, SIRT1 promoter was significantly activated in HaCaT cells cultured with conditioned medium from the Caco-2 cells treated with paramylon. Next, using an in vitro co-culture system, we showed that secretory factors from Caco-2 cells treated with 100 µg/mL of paramylon significantly activated the expression of endogenous SIRT1 in HaCaT cells (Fig. 3B), because paramylon can not pass through the membrane. In Fig. 2A and B, resveratrol (10 µM) was added directed to HaCaT cells and SIRT1 expression was evaluated. All these results demonstrated that paramylon activates SIRT1 in skin cells through the secretion of factors from paramylon-treated intestinal cells.

Further, we analyzed the functional categories of genes activated by paramylon from the viewpoint of gut–skin interaction (Table 1). Results showed that genes related to the functional categories of β-catenin-TCF complex assembly and secretion were significantly up- or down-regulated in Caco-2 cells upon the treatment with paramylon. β-catenin-TCF is known to be involved in the growth and maintenance of epidermal cells; therefore the results indicate that paramylon can activate epidermal cells by inducing the production of secretory factors from Caco-2 cells.

Skin SIRT1 is known to repair DNA damage induced by ultraviolet B irradiation via SIRT1-dependent activation of XPC/XPA (Chong et al. 2019). This study showed that orally ingested paramylon activates skin SIRT1 expression via the intestine, putatively by inducing the production of secretory factors that activate skin SIRT1, by stimulating gut–skin interaction. Additionally, the findings showed that paramylon repairs DNA damage and suppresses skin aging following oral ingestion. In subsequent studies, we aim to clarify the molecular basis of the activation of the gut–skin interaction by paramylon, and to test whether orally ingested paramylon can suppress skin aging, and further can achieve systemic anti-aging.

**Table 1:** Functional categories of genes activated by paramylon

Functional categories	P-value
β-catenin-TCF complex assembly	0.00014
Secreted	0.012
Collagen	0.14
Calcium channel activity	0.17

## Data Availability

All relevant data can be provided upon request.
